# Transcriptome Profiles of Nod Factor-independent Symbiosis in the Tropical Legume *Aeschynomene evenia*

**DOI:** 10.1038/s41598-018-29301-0

**Published:** 2018-07-19

**Authors:** Djamel Gully, Pierre Czernic, Stéphane Cruveiller, Frédéric Mahé, Cyrille Longin, David Vallenet, Philippe François, Sabine Nidelet, Stéphanie Rialle, Eric Giraud, Jean-François Arrighi, Maitrayee DasGupta, Fabienne Cartieaux

**Affiliations:** 1 LSTM, Univ. Montpellier, CIRAD, INRA, IRD, SupAgro, Montpellier, France; 2 Université de Montpellier, Place Eugène Bataillon, F-34095 Montpellier Cedex 5, France; 3 LABGeM, Génomique Métabolique, Genoscope, Institut François Jacob, CEA, CNRS, Univ Evry, Université Paris-Saclay, F-91057 Evry, France; 4 MGX, Univ. Montpellier, CNRS, INSERM, BioCampus, Montpellier, France; 5 Department of Biochemistry, University of Calcutta, Kolkata, 700019 India

## Abstract

Nod factors (NF) were assumed to be indispensable for the establishment of a rhizobium-legume symbiosis until the discovery that certain *Bradyrhizobium* strains interacting with certain *Aeschynomene* species lack the canonical *nodABC* genes required for their synthesis. So far, the molecular dialogue between Aeschynomene and its symbionts remains an open question. Here we report a time course transcriptional analysis of *Aeschynomene evenia* in response to inoculation with *Bradyrhizobium* ORS278. The NF-independent symbiotic process was monitored at five time points between bacterial infection and nodule maturity. The five time points correspond to three specific events, root infection by crack entry, nodule organogenesis, and the establishment of the nitrogen fixing process. During the third stage, about 80 NCR-like genes and eight symbiotic genes known to be involved in signaling, bacterial infection or nodulation regulation were highly expressed. Comparative gene expression analyses at the five time points also enabled the selection of genes with an expression profile that makes them promising markers to monitor early plant responses to bacteria. Such markers could be used in bioassays to identify the nature of the bacterial signal(s). Our data represent valuable resources for investigation of this Nod factor-independent symbiosis.

## Introduction

Legumes and cereals are among the earliest plants domesticated by human beings. Beans have been cultivated since the Neolithic in South America and lentils were already well known and consumed in Antiquity in Europe^1^. In addition to their use as human food, legumes such as alfalfa, clover and soybean have been widely used as forage since humans started breeding animals. Legumes have a beneficial effect on human health and nutrition thanks to their high protein content. Legumes also help improve soil fertility in crop rotations or intercropping systems. These two traits rely on the ability of legumes to biologically fix nitrogen. Legumes, in symbiosis with bacteria, collectively known as rhizobia, are able to convert atmospheric dinitrogen into ammonia, which is then assimilated by the host plant. Rhizobium-legume symbiosis is a win-win association in which bacteria fix nitrogen for the benefit of the plant in exchange for dicarboxylic acids resulting from photosynthesis. Nitrogen fixation takes place in a dedicated symbiotic organ, a nodule, which develops on the roots of the host plant and hosts the bacteria.

Two model legumes emerged in studies of biological nitrogen fixation in the 1990s. The need for two models was because the process of nodule organogenesis differs in *Medicago truncatula* and *Lotus japonicus*: in the former, nodules possess an active meristem throughout their life, which leads to an elongated organ, while in *L*. *japonicus*, meristematic activity is transient and gives rise to a spherical nodule. Apart from their meristematic nodule activity, *M*. *truncatula* and *L*. *japonicus* share a common bacterial infection process and a very similar genetic program recruited from the more ancient and widespread mycorrhizal symbiosis^2,3^.

The interaction between legumes and rhizobia relies on a sophisticated recognition system. Legumes first produce flavonoids that are perceived by rhizobia and induce the expression of *nod* genes involved in the synthesis and secretion of lipochitooligosaccharide signals (called Nod factors, NF). In turn, the perception and recognition of NFs by the host plant trigger a series of responses involved in nodule inception and infection. In most legumes, including model legumes, rhizobia invade plant tissues in an intracellular way through infection threads (IT) consisting in an invagination of the plasma membrane initiated at the tip of a deformed root hair^2,3^.

However, in about 25% of legumes found mainly in tropical and warm temperate areas, rhizobial invasion does not involve the formation of infection threads but an intercellular process^4^. In a few rare cases, the symbiotic interaction may even take place despite the absence of Nod factors. This is the case of some *Aeschynomene* species that are nodulated by photosynthetic *Bradyrhizobium* strains lacking the canonical *nodABC* genes required for NF synthesis^5^. This NF independent infection process is initiated when the root are penetrated through epidermal cracks at the lateral root emergence sites^6^. Subsequently, sub-epidermal cell death occurs that enables the propagation of the bacteria within the root endodermis. Finally, the infection of one or several cortical cells by direct endocytosis of rhizobia, triggers their division and the development of a nodule that becomes functional (i.e. able to fix atmospheric nitrogen) five days after inoculation^6^. Thanks to studies of the genetic diversity of *Aeschynomene* species that use an NF-independent process, *Aeschynomene evenia* has become a new model legume for the purpose of deciphering the molecular mechanisms of the NF-independent symbiosis^7^. Using this new model species, we recently demonstrated that two common symbiotic genes^8,9^ (*SYMRK* and *CCaMK*) and a cytokinin receptor involved in nodule organogenesis^10^ (*HK1*) were recruited for efficient nodulation in *A*. *evenia*^11^. Unexpectedly, the mechanisms also appeared to be conserved at the nodule maturation level. Indeed, although Aeschynomene are not close to the IRLC clade, some species - including *A*. *evenia* - use cysteine rich peptides similar to NCR to control bacterial differentiation in nitrogen fixing bacteroids^12^.

Therefore, the most enigmatic characteristic of Aeschynomene model system remains the absence of the need for NFs to induce the symbiosis. The plant and bacterial signal molecule(s) that control the establishment of the symbiosis between *A*. *evenia* and its symbiont remain to be identified. To advance our understanding of the initial steps of the interaction, we performed RNAseq analysis during a complete time course of the infection process starting as early as 6 hours post inoculation to 6 days post infection when the nodules have just become functional. The RNAseq data were confirmed by RTqPCR analyses for selected genes. Identification of differentially expressed genes (DEGs) at the different time points, including genes known to be involved in the symbiotic process of model legumes, was consistent with the general process of nodule organogenesis, development and functioning. Special focus was also placed on genes whose early stable induction following inoculation makes them suitable for a reporter gene strategy.

## Results and Discussion

### RNA sampling according to the symbiotic infection process

**Figure 1:**
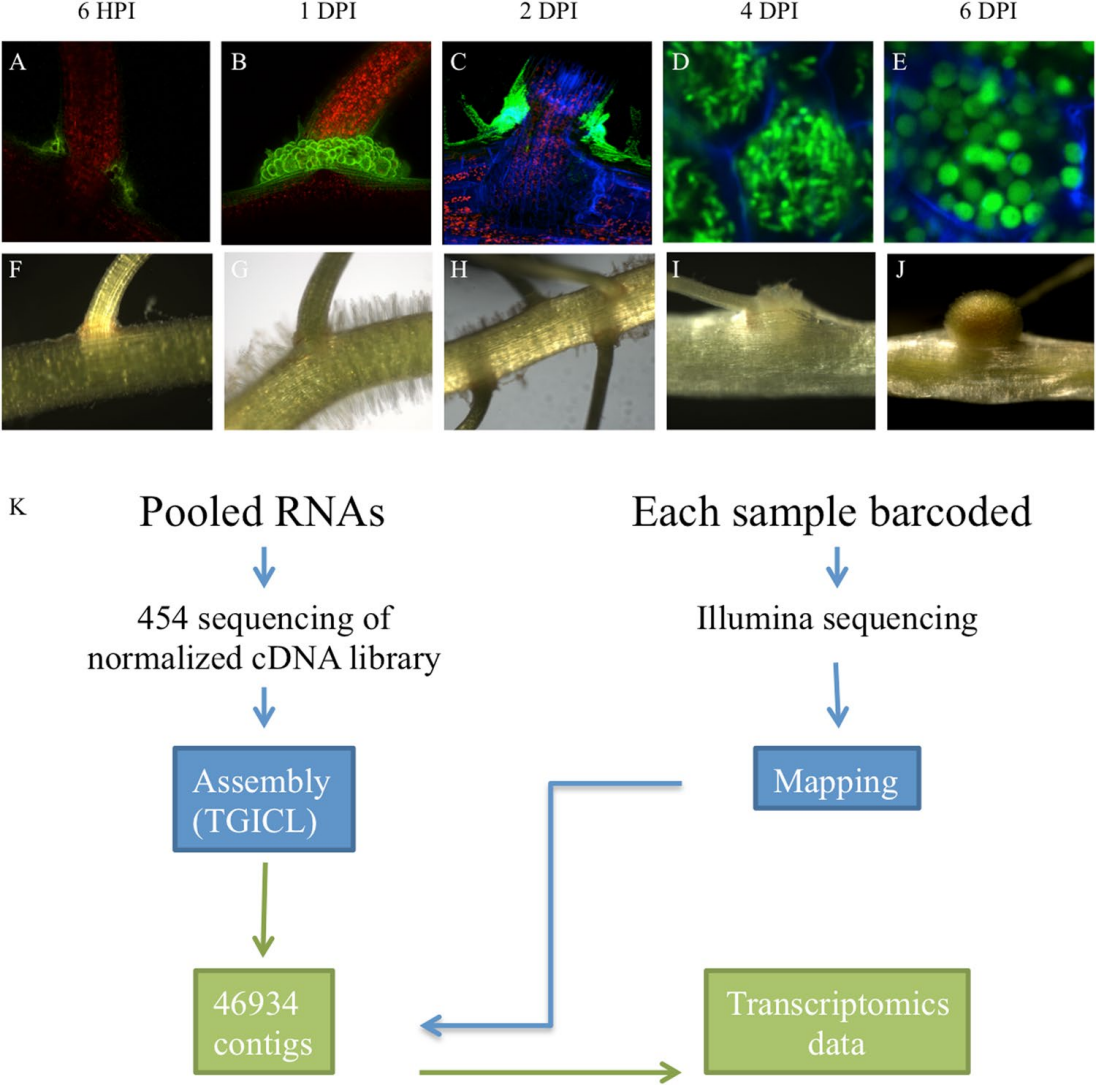
Experimental design and description of root samples collected at the different time points during the nodulation kinetics from non-inoculated roots (T0, control roots) to 6 days post inoculation (dpi) with *Bradyrhizobium* ORS278. (**A**–**E**) Confocal microscopy images of the root system inoculated with a GFP-tagged Bradyrhizobium strain to monitor the infection and differentiation processes. (**F**–**J**) Bright-field macroscope pictures of the roots. (**A**,**F**) Roots 6 hours post inoculation: the initial steps of perception. (**B**,**G**) Roots 1 dpi: colonization of an emerging lateral root; (**C**,**H**) roots 2 dpi: sub-epidermal infection; (**D**,**I**) roots 4 dpi: immature nodule colonized by undifferentiated bacteria; (**E**,**J**) roots 6 dpi: fixing nodule colonized by differentiated rhizobia, adapted from Arrighi *et al*.^7^. (**K**) RNAseq strategy: 454 sequences were clustered and assembled using TGICL to generate a transcriptome reference library. Illumina reads were mapped to 46,934 concatenated contigs of the transcriptome reference before statistical analyses were performed and differentially expressed gene patterns were obtained.

To decipher the molecular mechanisms underlying the NF-independent symbiotic process in *A*. *evenia* spp. *serrulata*, the accession used for the functional characterization of four symbiotic genes in Aeschynomene^11,12^, we prepared RNA samples from roots inoculated or not with *Bradyrhizobium* sp. ORS278. Roots were collected before inoculation (T0), 6 hours post-inoculation (hpi), 1 day post-inoculation (dpi), 2, 4 and 6 dpi. These time points correspond to the different steps of infection and nodule development already described in *A*. *evenia*^7^: we collected roots at 6 hpi to analyze early responses to infection before any macroscopic changes occurred, except bacterial colonization of lateral roots (Fig. 1A,F); as early as 1 dpi, dense colonization of the axillary root hairs surrounding the lateral roots was observed (Fig. 1B,G); at 2 dpi, a few cortical cells had been invaded and began to divide (Fig. 1C,H); at 4 dpi the nodule primordium was clearly visible (Fig. 1I) and cells infected with undifferentiated rod-shaped bacteria were visible (Fig. 1D); at 6 dpi, bacteria were differentiated into spherical bacteroids (Fig. 1E), the nodule was mature, as evidenced by the pink color due to the accumulation of leghemoglobin (Fig. 1J) and nitrogenase activity was detectable^6^.

### EST sequencing and clustering, sequence annotation and database creation

A normalized cDNA library from *A*. *evenia* tissues corresponding to non-inoculated roots, roots inoculated with *Bradyrhizobium* sp. ORS278 and collected 6 hpi, 2 dpi, 4 dpi, 7 dpi, mature nodules and leaves harvested 21 dpi, was pyrosequenced to obtain a reference set of transcripts. A total of 1,488,532 raw reads with an average length of 297 nucleotides corresponding to a total of 442 Mb were obtained (Table 1). The sequences were analyzed and clustered using a modified version of a pipeline called Expressed Sequence Tag treatment and investigation kit^13^ (ESTtik). A total of 46,934 *A*. *evenia* spp. *serrulata* EST contigs were assembled, of which 71% were blast annotated. The annotation results revealed that 29,120 contigs (61.3%) had significant matches in the TrEMBL database, 1,719 (3.61%) in the Nt database, 1,682 (3.54%) in the Nr database and 1,205 (2.53%) in the SWISS-PROT database. These *A*. *evenia* EST sequences are accessible on the SESAM web site: https://www.genoscope.cns.fr/agc/sesam.

**Table 1:** Raw data from Illumina and 454 sequencing.

	Samples	Number of reads	Number of bases	Mapped reads	Non-ambiguous mapping rate (%)
Illumina raw data	T0	52 300 676	5 230 067 567	48 067 215	91,91
	6HPI	51 082 419	5 108 241 867	46 906 291	91,82
	24HPI	48 800 573	4 880 057 300	44 793 759	91,79
	2DPI	55 930 717	5 593 071 700	51 898 744	92,79
	4DPI	61 361 611	6 136 161 133	51 898 744	84,58
	6DPI	57 676 234	5 767 623 367	53 456 518	92,68
	Total	327 152 229	32 715 222 933	297 021 271	90,79
Raw 454 data		1 488 532	442 226 657		

In parallel, the six different non-normalized cDNA libraries corresponding to the infection kinetic time points (T0, 6 hpi, 1 dpi, 2 dpi, 4 dpi, 6 dpi) were individually sequenced in triplicate using HiSeq2000 technology (Illumina Inc., USA). A total of 327,152,229 reads with a length of 100 nucleotides corresponding to 32,715 MB were obtained and mapped onto the contig clusters generated from the 454 sequencing data using the ssaha2 package (Table 1; Fig. 1K). The expression level of each assembled transcript sequence in different samples was measured using DESeq. 1 statistical analysis.

### Identification of differentially expressed genes

**Figure 2:**
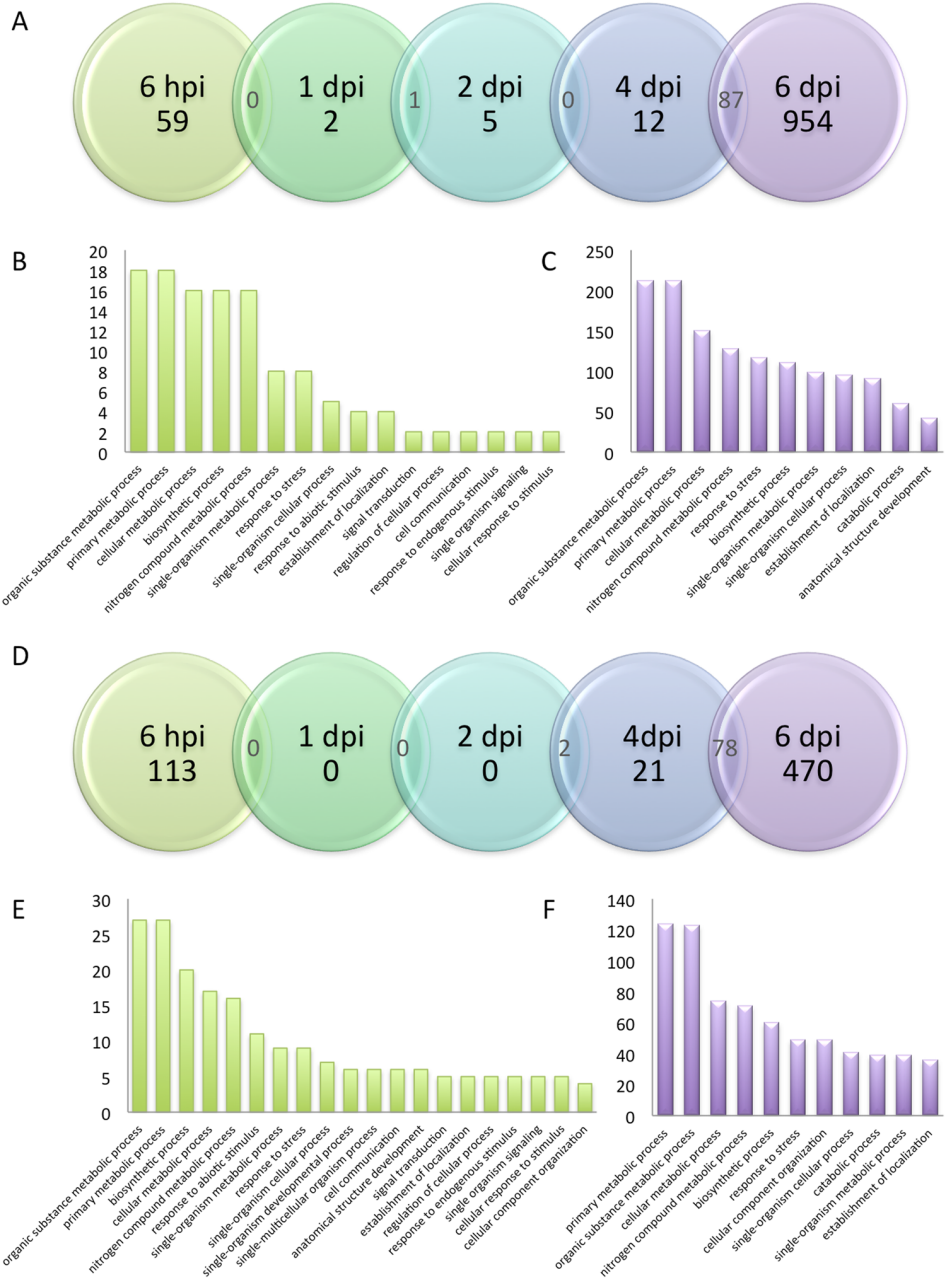
Differentially expressed genes. Venn diagram showing the genes found to be up-regulated (**A**) or down-regulated (**D**) during the time course of *Aeschynomene evenia spp*. *serrulata* inoculation with *Bradyrhizobium* ORS278. In both situations, DEGs were classified by gene ontology annotation for biological processes at 6 hpi (**B**,**E**) or 6 dpi (**C**,**F**) respectively. Overlaps between 2 adjacent time point conditions are also indicated. The details of the kinetics are the same as those described in Fig. 1.

Differentially expressed genes (DEG) in one sample compared to the non-inoculated root were defined using the following parameters: adjusted p-value < 0.05 and log2 fold change > 2. In total, 1276 genes were found to be up-regulated during the infection kinetics and 712 genes were found to be down regulated. Focusing on DEGs specific to each stage of the infection process (Fig. 2) led to the identification of 1,032 up-regulated genes (548 functionally annotated, Supplementary Table 1) and 604 down-regulated genes (448 functionally annotated, Supplementary Table 1).

Among the stage-specific DEGs, most were identified in nitrogen-fixing nodules (6 dpi), where 954 and 470 genes were found to be up- and down-regulated, respectively; and to a lesser extend in the initial step of the colonization (6 hpi, 59 and 113 genes up- and down-regulated, respectively). Only a few number of genes appeared to be specific to the intermediate time points (47 up- and 50 down-regulated genes for 1, 2 and 4 dpi combined; Fig. 2A). These observations were corroborated through hierarchical clustering analysis of the different time points. The two first (6 hpi and 1 dpi) and the two intermediate (2 and 4 dpi) time points appeared to cluster together, respectively, whereas the late point (6 dpi) corresponding to mature nodule behaved independently (Supplementary Fig. 1). We therefore focused our analyses on the early and late time points and performed a GO annotation for the biological processes involved (Fig. 2B,C,E and F).

#### Early responses

At 6 hpi, the majority of DEGs were involved in metabolic, biosynthetic and single organism processes (metabolic, cellular, signaling). These processes probably correspond to the induction of the signaling pathways leading to the shift from a developing root to nodule organogenesis.

**Figure 3:**
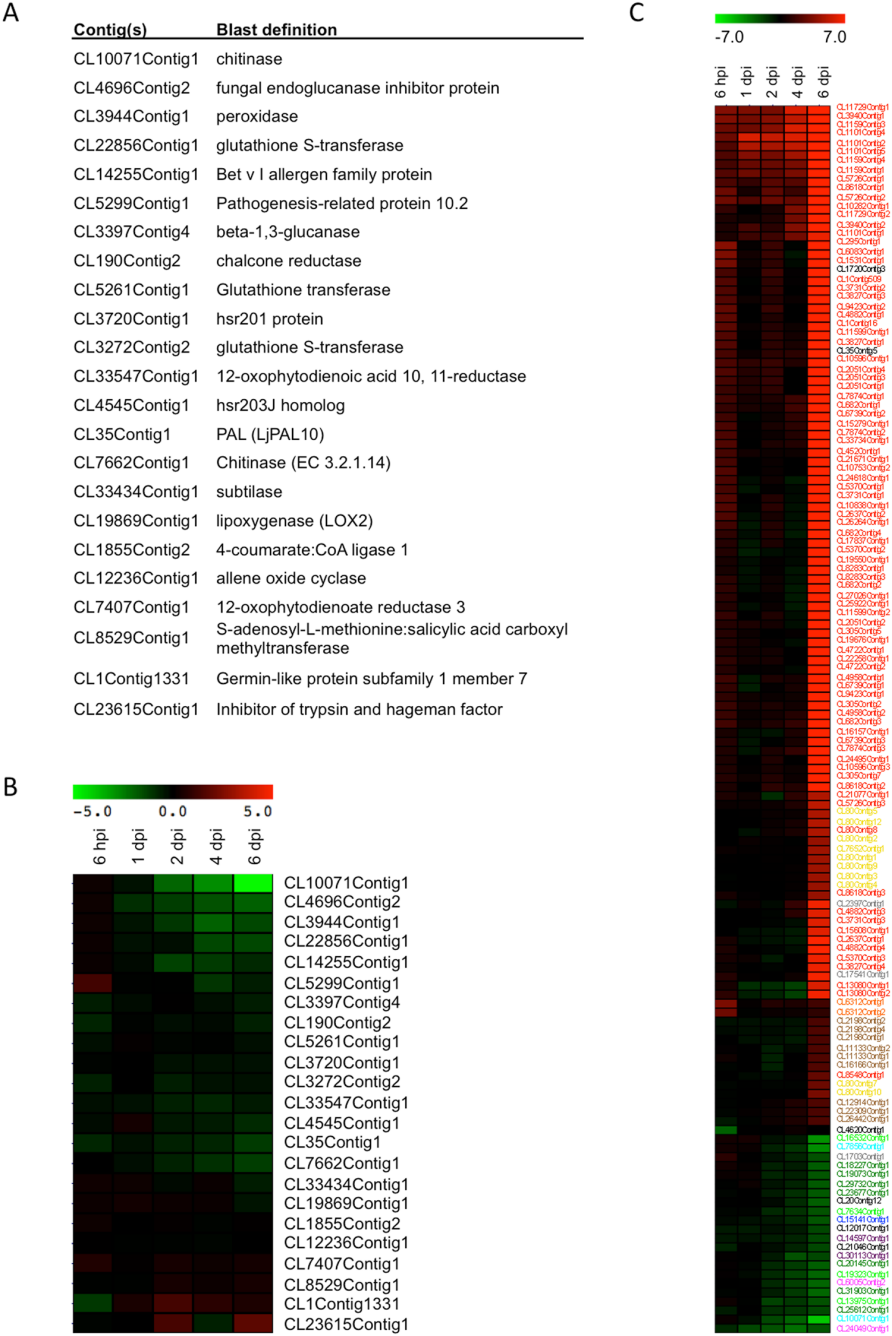
Heatmap of defense-related genes throughout nodulation kinetics. (**A**) The contig identification number and the BLAST definition of 23 genes known to be transiently up-regulated in response to symbionts in model legumes are presented in the table. (**B**) Behavior of the 23 corresponding orthologs in Aeschynomene. (**C**) Heatmap of differentially expressed defense-related genes throughout nodulation kinetics. Contigs corresponding to NCR are red, hypersensitive-induced response protein yellow, pathogenesis-related protein grey, MLO-like protein brown, isochorismatase orange, RLK protein dark green, disease resistance protein light green, LRR-containing protein purple, TIR-NBS disease resistance protein in pink, LysM domain containing protein in blue and chitinase in cyan. Red indicates up-regulation and green indicates down-regulation. Chroma color from green to red indicates Log2 (fold change) from less to more.

A smaller number of DEGs were linked to biological processes corresponding to “Response to Stress” and “Abiotic Stimuli”. More detailed analysis of the genes specifically induced at 6 hpi highlighted two genes encoding isochorismatases (CL6312Contig1 & 2; Supplementary Table 1; Fig. 3C). Isochorismatases are able to deplete isochorismate, the precursor in the synthesis pathway of the main plant defense hormone, salicylic acid^14^. It was recently reported that such enzyme activity was used by both an oomycete and a fungal pathogen to interfere with salicylic acid signaling and to reduce defense responses^15^. In order to confirm this observation, we focused on several defense-related genes previously shown to be transiently induced in model legumes such as medicago^16,17^, lotus^18^ or soybean^19^. First, we identified the corresponding Aeschynomene orthologs (Fig. 3A) and then analyzed their expression profiles. As can be seen in Fig. 3B, none of them appeared significantly induced throughout the nodulation kinetics (adjusted p-value < 0,05). In agreement with this general absence in defense response, many other putative defense signaling genes were found to be down regulated (Fig. 3C) e.g. LRR-containing proteins (2 genes), receptor-like-kinases (7 genes), TIR-NBS disease resistance protein (2 genes) and a LysM domain containing protein (2 genes). These results suggest that the plant defense mechanisms are muted, at least in the early stages of the interaction. Such trade-offs between the symbiotic infection process and defense mechanisms are well documented^20^.

In sharp contrast, other candidate genes for signal transduction or gene regulation were induced as early as 6 hpi. These included 1 serine/threonine kinase, 1 MYB and 4 other transcription factors, as well as 3 genes encoding two component response regulators. Theses latter have been shown to be associated with cytokinin signaling, the hormone associated with nodule organogenesis in legumes^21^. These observations suggest that they all play an important role in the shift from root development to nodule organogenesis. The analysis of the genes specifically involved in the symbiotic process is reported in the “Behavior of symbiotic genes in the *A*. *evenia* RNA-seq database” section below.

#### Late responses

As mentioned in the “Early responses” section above, among the 1,276 up-regulated genes, 954 were specific to the 6 dpi time point (Fig. 2A) at which time the nodule is functional and able to fix nitrogen. A functional categorization based on gene ontology (GO) was conducted on the 593 contigs that were annotated. As expected, and as shown in Fig. 2C, six out of the 11 GO groups were related to metabolic or catabolic processes. A fine analysis of the “Response to Stress” GO group enabled the identification of 84 nodule-cysteine-rich (NCR) peptides encoding genes as those with the highest induction levels at 6 dpi (as manually annotated in Supplementary Table 1). NCR-like peptides have already been shown to be implicated in the differentiation of bacteroids in Aeschynomene nodules, and some have already been shown to be highly expressed in this organ^12^. Among the previously identified 82 NCR-like genes^12^, 73 were highly expressed at 6 dpi. A more detailed analysis enabled the identification of an additional 11 NCR-like encoding genes (Supplementary Table 1). Interestingly, in the “Response to Stress” GO group, several defense-related encoding genes, including 1 PAL, 2 PR proteins; 11 hypersensitive-induced response proteins and 9 MLO-like proteins, were induced in the 6 dpi time point (Fig. 3C). Taken together, these data suggest that Aeschynomene not only uses NCRs but also several other defense genes in mature nodules to keep its symbionts under control. This situation contrasts with the early time points where defense reactions appeared to be attenuated. Interestingly, one of the most highly induced genes at the 6 dpi time point corresponds to a NPR1 homolog (CL1720Contig3). NPR1 is a transcriptional regulator required for defense gene induction in response to salicylic acid^22^. Although we were able to identify 5 NPR1 annotated genes in our database, only the CL1720Contig3 gene appeared specifically induced in nodules (Fig. 3C). It is therefore tempting to speculate that many of the defense-related genes, and especially the NCR genes, that are highly induced in nodules could be regulated through this regulator. A reverse genetic approach such as the one we already performed for DNF1^12^ would help to test this hypothesis.

The mature nodules also had the highest number of down-regulated genes. Among the 470 genes, 283 were annotated and almost 50% of them (201) were involved in cell wall dynamics (Supplementary Table 1) including structural proteins (hydroxyproline-rich glycoprotein also known as extensin, 82 genes) or modeling enzymes (polygalacturonase, 6 genes; pectate lyase, 6 genes; pectin esterase, 13 genes). It should be noted that the time point 6 dpi corresponds to the isolated mature module stage (with limited growth and cell divisions) compared to non-inoculated roots (young tissue under strong elongation driven by cell division and cell wall extension). Therefore, it seems logical that all these genes linked with cell-wall dynamics are down regulated (Supplementary Fig. 2).

### Validation of differentially expressed genes using qRT-PCR

**Figure 4:**
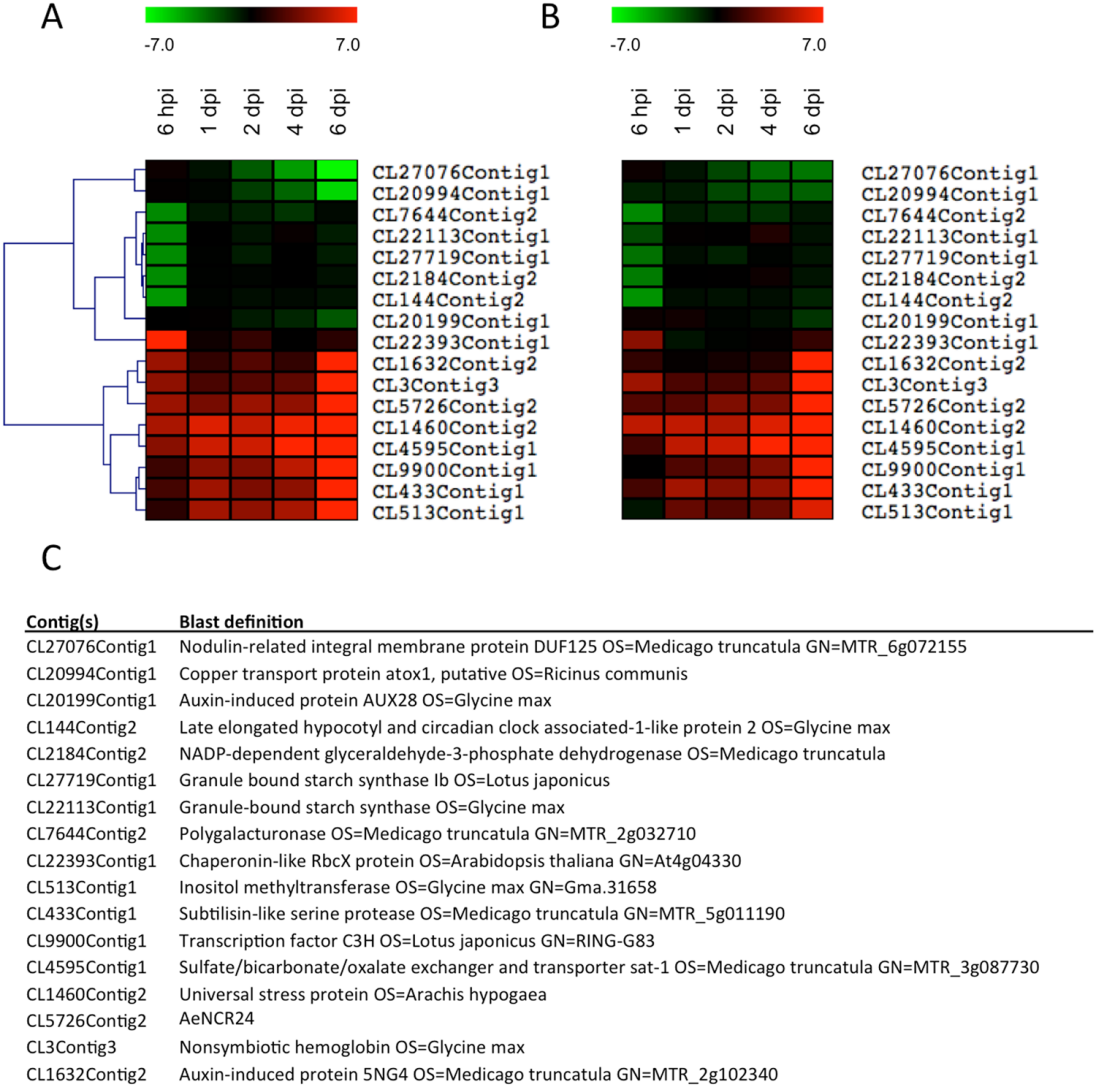
Heat maps of 17 differentially expressed genes with contrasted behavior during the infection process obtained from RNAseq data (**A**) and from real time quantitative RT-PCR (qPCR) (**B**). The contig identification number and the BLAST definition of these 17 genes are presented in the table (**C**). Three biological replicates were performed for each RT-qPCR validation; the results are expressed as means. The elongation factor one alpha (EF1æ) gene was used as internal control. All results are expressed as Log2 fold change. The color scale corresponding to the expression fold change is presented above panels (A and B). Clustering was performed with the MultiExperiment Viewer 4.8 by hierarchical clustering of all genes and samples using Manhattan distance calculations.

As a first step in validating our RNAseq data, we focused on 17 genes among the most up- and down-regulated in *A*. *evenia* during the first step of the interaction (6 hpi) or in the nodule tissue (6 dpi). Their corresponding messenger-RNA accumulation was examined in three biological replicates using qRT-PCR to confirm the reliability of the RNA-seq analysis (Fig. 4A–C). It is noteworthy that among the most up-regulated genes at 6 dpi, we found genes encoding a nodulin ortholog, leghemoglobin and a nodule rich cysteine peptide^12^ (AeNCR24) known to be expressed in nodules of model legumes. All the orthologs of the candidate genes selected for validation of RNA-seq data had a similar expression profile in *M*. *truncatula* and in *Glycine max*^23^. The fold changes calculated from qRT-PCR data are expressed in log2 to facilitate comparison with RNA-seq data. For the same reason, expression profiles, visualized as heatmaps, were subjected to hierarchical clustering using MEV software^24^ (MeV 4.8.0 software). The results showed that the gene expression patterns evaluated by qRT-PCR (Fig. 4B) were consistent with the RNAseq data (Fig. 4A), evidence that our RNA-Seq analysis was robust.

### Behavior of symbiotic genes in the *A*. *evenia* RNA-seq database

**Figure 5:**
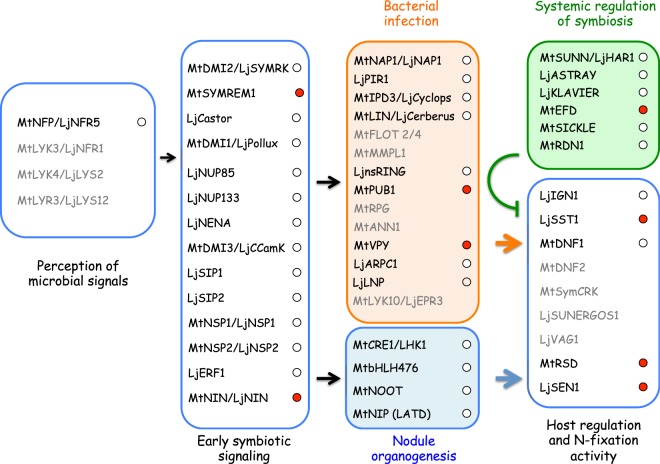
Schematic representation of the gene cascades in symbiotic signaling symbiotic genes identified in the *Medicago truncatula* and *Lotus japonicus* genomes were classified according to their putative roles in root nodule symbiosis. The genes identified in *A*. *evenia spp*. *serrulata* are black and the symbiotic genes for which no transcripts were detected are grey. Regulation of transcription during the nodulation kinetics is in the circle: in white for constitutive expression; in red for up-regulation; in green for down-regulation. Adapted from Chaintreuil *et al*.^25^.

We searched our database for genes reported to be involved in all the different steps in the symbiotic process in model legumes. Among the 52 symbiotic genes characterized in *M*. *truncatula* and *L*. *japonicus* we checked (Fig. 5; Supplementary Table 2), we were able to identify 39 orthologs in *A*. *evenia*. The list of present or absent symbiotic genes is consistent with previous Illumina data used to develop molecular markers in another *A*. *evenia* accession of the spp. *evenia*^25^. The symbiotic genes for which orthologs were identified in both *A*. *evenia* accessions are required in all the different steps of the nodulation process: from the perception of the rhizobium to the induction of the signaling pathways that control bacterial infection and nodule organogenesis and finally in local and systemic regulation of nitrogen fixation (Fig. 5). The distinctive feature and advantage of the present study is monitoring the expression profiles of these symbiotic genes throughout nodulation kinetics. We found strong induction of expression in eight of them (*MtSYMREM1*, *MtNIN/LjNIN*, *MtPUB1*, *MtVPY*, *MtEFD*, *LjSST1*, *MtRSD*, *LjSEN1*) throughout nodule development, comparable to the behavior reported in *M*. *truncatula* and *L*. *japonicus* (for a review see^2,3^). MtSYMREM1 is a symbiotic receptor-binding protein that interacts with the *bona fide* receptors MtLYK3/LjNFR1, MtNFP/LjNFR5 and MtDMI2/LjSYMRK^21^. Expression pattern of *MtSYMREM1* and the phenotypes of loss-of-function mutants suggest a role for this gene in bacterial signal perception during the initial stages of infection and throughout nodule development^26^. The expression profile of the *SYMREM1* ortholog in *A*. *evenia* differs slightly from the expression profile of *MtSYMREM1*^25^: no induction was observed during the very first steps of bacterial perception by the host plant; the induction of expression *AeSYMREM1* started at 4 dpi and peaked at 6 dpi when nodules were fully functional (Supplementary Fig. 3). We observed the same time shift in induction for *NIN*, encoding a transcription regulator that is essential for nodulation^27^, *MtPUB1*, encoding an E3 ubiquitin ligase that interacts with the NF receptor MtLYK3^28^ and *MtVPY*, a common symbiotic gene^29^. The fact that the crack-entry infection process bypasses epidermal responses to rhizobia could explain why the induction of *SYMREM1*, *NIN*, *PUB1* and *VPY* orthologs during the early steps of nodulation is not observed in *A*. *evenia*, whereas the high level of transcription in nodules for those genes is conserved between model legumes and *A*. *evenia*. Nodule specific induction of expression was also conserved in the ortholog of *MtEFD* and in the orthologs of *LjSST1*, *MtRSD* and *LjSEN1*, the former being involved in systemic regulation of symbiosis and the latter in host regulation and nitrogen-fixation activity (Fig. 5).

In addition to this panel of symbiotic genes whose expression profile is at least partly conserved between model legumes and *A*. *evenia*, we also identified nine orthologs of symbiotic genes (*LjNENA*, *MtNSP1/LjNSP1*, *MtNSP2/LjNSP2*, *LjSIP1*, *LjERF1*, *MtLIN/LjCERBERUS*, *MtNOOT*, *LjnsRING*, *MtDNF1*) whose expression was not significantly affected during the symbiotic process contrary to what has been reported in *M*. *truncatula* or *L*. *japonicus*^30–40^. In the corresponding model legumes, five of the genes (*MtNSP1/LjNSP1*, *MtNSP2/LjNSP2*, *LjSIP1*, *LjERF1*, *MtNOOT*) display a moderate, and sometimes transitory (*LjSIP1*, *LjERF1*), increase in their transcription level under symbiotic conditions, which could explain why we failed to detect the modulation of transcription of these genes in our dataset. There were still four symbiotic genes (*LjNENA*, *MtLIN/LjCERBERUS*, *LjnsRING* and *MtDNF1*) whose expression significantly increased during nodule development in model legumes but not in *A*. *evenia*. *DNF1* encodes the SPC22 subunit of a nodule-specific signal peptidase complex required for maturation of NCR peptides. *DNF1* is the only one of the subset of four nodule-expressed genes that have undergone RNAi experiments and whose role in nodulation was demonstrated in *A*. *evenia*^12^. It is even more surprising not to find an expression profile similar to that observed in *M*. *truncatula*. Still, no straightforward hypothesis can be proposed concerning the absence of *DNF1* up-regulation in *A*. *evenia* nodules. In the case of *LjNENA*, *LjnsRING* and *MtLIN/LjCERBERUS*, three genes required for the initiation or growth of infection threads, the intercellular infection process of *Aeschynomene* species could once again explain the differences in transcriptional regulation observed between *A*. *evenia* and temperate model legumes in which rhizobia use root hair infection threads to invade plant tissues in an intracellular way^6,30,37–39^. The phenotype of *nena* mutants is particularly relevant. No root hair curling or root hair infection threads were observed in *nena* mutants, but a few infected nodules appeared 21 days post infection^30^. Detailed microscopic analyses of the mutants revealed that the rhizobial infection mode in *nena* mutants carried the hallmarks of crack entry, the infection process at work in *A*. *evenia*. This is additional evidence that some, if not all, epidermal responses to rhizobia are bypassed during the crack entry infection process.

Concerning the 13 symbiotic genes for which transcripts were not detected in *A*. *evenia* transcriptomes, sequencing of *A*. *evenia* genome is currently underway and will enable us to clarify if these genes do exist in the *A*. *evenia* genome but are not, or only very weakly, expressed in our conditions. Nevertheless, as previously observed in *A*. *evenia* spp. *evenia*^7^, it is significant that nine out of thirteen non-expressed genes in *A*. *evenia* (*MtLYK3/LjNFR1*, *MtLYK4/LjLYS2*, *MtLYR3/LjLYS12*, *MtLYK10/LjEPR3*, *MtFLOT2*, *MtFLOT4*, *MtMMPL1*, *MtRPG*, *MtANN1*) are either involved in NF perception or IT formation and growth, two processes that are not involved in the *A*. *evenia-Bradyrhizobium* ORS278 symbiosis^41–48^. The four other genes missing in our data set are required for nodule functioning^49,50^ (*LjSUNERGOS1*, *LjVAG1*) or control of immunity^51,52^ (*MtDNF2*, *MtSymCRK*).

In conclusion, the BLAST search performed using the sequence of symbiotic genes characterized in *M*. *truncatula* and *L*. *japonicus* enabled the identification of 39 orthologs in *A*. *evenia* among which 13 display different expression profiles from those observed in the two model legumes. These observations demonstrate that specific mechanisms occur during the NF-independent symbiosis even if the common signaling pathway was shown to be partly recruited in *A*. *evenia*^11^.

### Toward the identification of molecular markers for the search for *Bradyrhizobium* signal molecule(s)

Since the discovery of an NF-independent symbiosis in *Aeschynomene* species, the major challenge has been the elucidation of the first steps of the molecular dialogue between the plant and its symbionts. On the bacterial side, the signal molecule(s) used by *Bradyrhizobium* ORS278 to interact with its host plant in a NF-independent way remain(s) to be identified. The main obstacle to achieving this goal is the absence in Aeschynomene of an early easy to monitor symbiotic response to inoculation, like the root hair curling observed in model legumes in response to NFs. The use of molecular markers of the early stages of interaction as a bioassay appeared to be a good alternative to get round this problem. In fact such strategy was originally set up to purify Myc signals using a transgenic line of *M*. *truncatula* carrying a fusion between the promoter region of the early nodulin gene *MtENOD11* and the *GUS* reporter gene^53^. We attempted to use this bioassay but our results demonstrated that p*MtENOD11* is not activated during the early stages of the interaction of Aeschynomene with photosynthetic *bradyrhizobia* strains^54^.

RNAseq data will probably be able to provide better markers of early symbiotic stages. Initially, we were particularly concerned about the expression profile of the ortholog of well-characterized symbiotic genes such as *NIN*. NIN is a transcription regulator that is essential for nodulation but not required for arbuscular mycorrhizal (AM) symbiosis^27^. Its loss of function inhibits nodule organogenesis and bacterial infection and its expression is dramatically induced in the root epidermis and in the cortex during the infection process in both *M*. *truncatula* and *L*. *japonicus*^27,55^. The rapid induction of *NIN* expression in response to rhizobia inoculation in the two model legumes is conserved in actinorhizal plants that form root nodule symbiosis with *Frankia*^56^. Unfortunately, in *A*. *eveni*a, the induction of the putative ortholog of *NIN* significantly starts with nodule inception (Supplementary Fig. 3). This does not preclude conserved symbiotic function but the gene is apparently not a suitable candidate for a promoter::reporter gene construct capable of monitoring early plant responses to its symbionts.

Other candidates emerged from the RNAseq data according to their expression profiles, like those illustrated in Fig. 4. We focused on genes that displayed early and stable induction during the complete nodulation kinetics as observed in RNAseq data (Fig. 4A) and confirmed by RT-qPCR (Fig. 4B). Blast search revealed three contigs that appeared not to be related to symbiotic signaling processes: CL4595Contig1, CL1460Contig2 and CL5726Contig2 (Fig. 4C). Three candidates were retained: CL9900Contig1, which encodes a transcription factor; CL513Contig1, which shares homology with an inositol-O-methyltransferase, a protein involved in abiotic stress tolerance in *Glycine max*^57^ and CL433Contig1, which encodes a subtilase whose putative homolog in *L*. *japonicus*, *SbtM1*, is an early molecular marker of arbuscular mycorrhiza^58^. Silencing of *SbtM1* confirmed the role of subtilase during the fungal infection process^57^. Amongst other modes of action, plant subtilases are believed to weaken cell-cell connections by degrading structural protein in the apoplastic space and may thus be required for elongation of the fungal hyphae in the intercellular space. This putative role of subtilase in cell wall loosening could also be at play during the intercellular infection process in Aeschynomene. Taken together, these lines of evidence strongly support the choice of CL433Contig1 for the development of a bioassay. We will take advantage of genomics data currently being produced for *A*. *evenia* to identify the promoter sequences of CL513Contig1 and CL433Contig1 to develop a bioassay consisting in a fusion between their promoter regions with a *GUS::GFP* cassette. In parallel, it would be interesting to address the role of these new symbiotic genes, especially CL433Contig1, in silencing experiments.

In conclusion, the data generated by this RNAseq analysis represent an important advance in the understanding and decryption of the NF-independent symbiotic process. They also contain promising new genes to use as reporter markers to better characterize the nodulation process and to enable the identification of the signal molecule(s) used by *Bradyrhizobium* ORS278 to interact with *A*. *evenia*.

## Methods

### Plant and Bacterial Strains

*A*. *evenia* spp. *serrulata* species used in this study corresponds to line IRFL6945^7^ (USDA). Rhizobial inoculations and growth conditions for plants are detailed in^11^. Briefly, seeds were scarified with concentrated (95%) sulfuric acid and surface sterilized with 3% sodium hypochlorite. Germination was induced on 0.8% water agar plates for one day in a 34 °C chamber in the dark. One day-old seedlings were transferred to test tubes filled with BNM medium. Plants were grown in a 28 °C growth chamber with a 16 h/8 h light regime and 70% humidity. One week later, the plants were inoculated with 1 ml of a 5 day-old *Bradyrhizobium* sp. ORS278 strain^59^ washed and with the optical density at 600 nm adjusted to 1. Root tissues were collected from non-inoculated samples just before inoculation. Six hours post-inoculation (hpi), and 1, 2, 4, 6 and 7 days post-inoculation (dpi), root sections corresponding to the nodulation inception zone (1 to 2 cm long section bellow the collet) were harvested. Young developing leaves and mature nodules were collected from 3-week-old *A*. *evenia* plants.

### Total RNA isolation

Total RNA was extracted from roots using the SV total RNA Isolation System (Promega) and quantified using a NanoDrop ND-1000 spectrophotometer. We used a pool of 24 plants for each time point. The integrity of the RNA samples was checked using an Agilent 2100 Bioanalyzer according to the manufacturer’s instructions.

### 454 and Illumina sequencing

(GATC Biotech, Mulhouse, France) and then used for 454 sequencing (Roche). The sequences obtained were analyzed using a pipeline called Expressed Sequence Tag treatment and investigation kit^13^ (ESTtik). This pipeline enabled editing and assembling of the input cDNA sequences in a non-redundant data set.

Six mRNA libraries corresponding to non-inoculated roots and roots inoculated at 6 hpi, 1, 2, 4 and 6 dpi, were built and sequenced at the MGX platform (Montpellier Genomix, *Institut de Genomique Fonctionnelle*, Montpellier France). The RNA libraries were constructed using the TruSeq stranded mRNA library construction kit (Illumina Inc., USA). The quantitative and qualitative analyses of the library were carried on Agilent_DNA 1000 chip and qPCR (Applied Biosystems 7500, SYBR Green). RNA was sequenced using the Illumina SBS (sequence by synthesis) technique on a Hiseq2000 in single read 100 nt mode. Image analysis, base calling and quality filtering were performed using Illumina software.

### RNAseq: short read counting method

The complete transcriptomic high-throughput sequencing data were analyzed with the TAMARA bioinformatic pipeline (Transcriptome Analyses based on MAssive sequencing of RnAs; Cruveiller S., unpublished) currently implemented on the Microscope platform^60^. The pipeline is a “Master” shell script that launches the different parts of the analysis and checks that all tasks are completed without error. We first assessed RNA-Seq data quality including options such as read trimming or the use of merging/split paired-end reads. Reads were then mapped onto the EST contigs of *A*. *evenia spp*. *serrulata* with the SSAHA2 package^61^. We minimized the false positive discovery rate by selecting only reliable alignments from SAM-formatted files using SAMtools^62^ (v.0.1.8). The number of reads matching each EST contig was then calculated with an enhanced version of the coverageBed software from the BEDtools suite^63^. Reads matching several genomic objects were weighted by the number of objects matched so as to keep the total number of reads in the library constant. Finally, the Bioconductor-DESeq package^64^ (v1.4.1) was used with default parameters for the analysis of raw count data and to determine whether expression levels differed significantly between conditions. The R script described in Supplementary Method 1 was used to select the up- or down-regulated gene sets specific for each time lapse point.

### qPCR analysis

One hundred nanograms of total RNA per sample were reverse transcripted using SuperScriptII reverse transcriptase (Invitrogen) and oligo(dT)12–18. Real time qPCR was performed using the Brilliant II SYBR® Green QPCR Master Mix (Agilent Technologies). Primer sequences are listed in Supplementary Table 3. The Real Time SYBR® Green cycling PCR program on a Stratagene MX3005P (Agilent Technologies) was as follows: one cycle at 95 °C for 10 min, 40 cycles at 95 °C for 10 s, 60 °C for 30 s and 72 °C for 30 s, ending with one cycle at 95 °C for one min, 55 °C for 30 s, and 95 °C for 30 s. Expression levels were normalized with the *AeEF1æ* reference gene. Three independent biological replicates were performed for each experiment and two qPCR technical replicates were performed for each biological replicate. The efficiency-corrected comparative quantification method was used to quantify the expression of genes of interest^65^.

### Data Availability

The reference transcriptome and the raw FASTQfiles for the 18 libraries were respectively deposited on NCBI under the bioprojects PRJNA396532 and PRJNA399681 with the accession numbers: SRR5983062, SRR5983061, SRR5983060, SRR5983059, SRR5983066, SRR5983065, SRR5983064, SRR5983063, SRR5983069, SRR5983067, SRR5983070, SRR5983068, SRR5983072, SRR5983071, SRR5983073, SRR5983074, SRR5983076, SRR5983075.

## Electronic supplementary material

Supplementary Method 1

Supplementary Figure 1

Supplementary Figure 2

Supplementary Figure 3

Supplementary Table 1

Supplementary Table 2

Supplementary Table 3
