# Supplementary material for: Transcriptome Profiles of Nod Factor-independent Symbiosis in the Tropical Legume Aeschynomene evenia
Source: Sci Rep. 2018 Jul 19;8:10934. doi: 10.1038/s41598-018-29301-0 (PMC6053390; doi:10.1038/s41598-018-29301-0)
Supplement: Supplementary file 1 — Supplementary Method 1 [file 41598_2018_29301_MOESM1_ESM.pdf]

Supplementary Method 1

Transcriptome Profiles of Nod Factor-independent Symbiosis in the Tropical Legume *Aeschynomene evenia*

Djamel Gully, Pierre Czernic, Stéphane Cruveiller, Frédéric Mahé, Cyrille Longin, David Vallenet, Philippe François, Sabine Nidelet, Stéphanie Rialle, Eric Giraud, Jean-François Arrighi, Maitrayee Das Gupta and Fabienne Cartieaux

Supplementary Method 1: The R script used to select the up- or down-regulated gene sets specific for each time lapse point.

Supplementary Method 1: R script used to select the up- or down-regulated gene sets specific for each time lapse point.

```
library(tidyverse)

## ------------------------------- Variables ------------------------------- ##

setwd("~/Science/Projects/Sesam/data/")
input_files <- c("DESeq_AES14.txt")
min_fold <- 2.0
max_p <- 0.05
hours <- c(6, 24, 48, 96, 144)  ## elapsed time

for (input_file in input_files) {

  ## ------------------------------- Data ----------------------------------- ##

  ## raw table
  d <- read_tsv(input_file)

  ## reorganized data (elapsed time as a variable)
  d1 <- d %>%
     gather("variables", "values", -id) %>%
     separate(variables, c("metrics", "elapsed"), sep = "_") %>%
     filter(metrics %in% c("log2foldchange", "padj")) %>%
     mutate(elapsed = sub("PI.T0", "", elapsed)) %>%
     spread(metrics, values) %>%
     mutate(t1 = as.integer(sub("[DH]", "", elapsed))) %>%
     mutate(t2 = sub("[0-9]+", "", elapsed)) %>%
     mutate(t2 = as.integer(if_else(t2 == "H", 1, 24))) %>%
     mutate(elapsed = t1 * t2) %>%
     select(-t1, -t2) %>%
     arrange(id, elapsed) %>%
     group_by(id)


  ## ************************************************************************* ##
  ##         ##
  ##                  any gene: up-or down-regulated          ##
  ##         ##
  ## ************************************************************************* ##

  ## all up-regulated genes (when compared to time zero)
  output_file <- sub(".txt", "_upregulated_any_condition.tsv", input_file)
  d1 %>%
     filter(log2foldchange >= min_fold & padj <= max_p) %>%
     ungroup() %>%
     select(id) %>%
     distinct(id) %>%
     left_join(d, by = "id") %>%
     write_tsv(output_file)

  ## all down-regulated genes (when compared to time zero)
  output_file <- sub(".txt", "_downregulated_any_condition.tsv", input_file)
  d1 %>%
     filter(log2foldchange <= -min_fold & padj <= max_p) %>%
     ungroup() %>%
     select(id) %>%
     distinct(id) %>%
     left_join(d, by = "id") %>%
     write_tsv(output_file)


  ## ************************************************************************* ##
```

```
##         ##
##              single condition: up- and down-regulated          ##
##         ##
## ************************************************************************ ##

## select genes that are up-regulated in one condition, and stable or
## down-regulated in all other conditions

## with any(), filtering operates per line (hours must be true for
## that line) but retains the whole group.

for (i in 1:length(hours)) {
    ## up-regulated
    output_file <- sub(".txt",
                    paste0("_upregulated_", hours[i], "h_only.tsv"),
                    input_file)
    d1 %>%
        filter(any(elapsed == hours[i] & log2foldchange >= min_fold & padj <= max_p)) %>%
        filter(!any(elapsed != hours[i] & log2foldchange >= min_fold & padj <= max_p)) %>%
        ungroup() %>%
        select(id) %>%
        distinct(id) %>%
        left_join(d, by = "id") %>%
        write_tsv(output_file)

    ## down-regulated in one condition (< -2), and stable or
    ## up-regulated in all other conditions !(< -2)
    output_file <- sub(".txt",
                    paste0("_downregulated_", hours[i], "h_only.tsv"),
                    input_file)
    d1 %>%
        filter(any(elapsed == hours[i] & log2foldchange <= -min_fold & padj <= max_p)) %>%
        filter(!any(elapsed != hours[i] & log2foldchange <= -min_fold & padj <= max_p)) %>%
        ungroup() %>%
        select(id) %>%
        distinct(id) %>%
        left_join(d, by = "id") %>%
        write_tsv(output_file)
}


## ************************************************************************ ##
##         ##
##              double condition: up- and down-regulated          ##
##         ##
## ************************************************************************ ##

## select genes that are up-regulated in two consecutive conditions,
## and stable or down-regulated in all other conditions

for (i in seq(1, length(hours) - 1)) {
    conditions <- c(hours[i], hours[i+1])
    ## up-regulated
    output_file <- sub(".txt",
                    paste0("_upregulated_", hours[i], "-", hours[i+1], "h_only.tsv"),
                    input_file)
    d1 %>%
        filter(any(elapsed == hours[i] & log2foldchange >= min_fold & padj <= max_p)) %>%
        filter(any(elapsed == hours[i+1] & log2foldchange >= min_fold & padj <= max_p)) %>%
        filter(!any(!(elapsed %in% conditions) & log2foldchange >= min_fold & padj <= max_p)) %>%
        ungroup() %>%
        select(id) %>%
        distinct(id) %>%
        left_join(d, by = "id") %>%
```

```
        write_tsv(output_file)

    ## down-regulated in two conditions (< -2), and stable or
    ## up-regulated in all other conditions !(< -2)
    output_file <- sub(".txt",
                    paste0("_downregulated_", hours[i], "-", hours[i+1], "h_only.tsv"),
                    input_file)
    d1 %>%
        filter(any(elapsed == hours[i] & log2foldchange <= -min_fold & padj <= max_p)) %>%
        filter(any(elapsed == hours[i+1] & log2foldchange <= -min_fold & padj <= max_p)) %>%
        filter(!any(!(elapsed %in% conditions) & log2foldchange <= -min_fold & padj <= max_p)) %>%
        ungroup() %>%
        select(id) %>%
        distinct(id) %>%
        left_join(d, by = "id") %>%
        write_tsv(output_file)
}


## ************************************************************************ ##
##          ##
##               triple condition: up- and down-regulated          ##
##          ##
## ************************************************************************ ##

## select genes that are up-regulated in three consecutive conditions,
## and stable or down-regulated in all other conditions

for (i in seq(1, length(hours) - 2)) {
    conditions <- c(hours[i], hours[i+1], hours[i+2])
    ## up-regulated
    output_file <- sub(".txt",
                    paste0("_upregulated_", hours[i], "-",
                        hours[i+1], "-", hours[i+2], "h_only.tsv"),
                    input_file)
    d1 %>%
        filter(any(elapsed == hours[i] & log2foldchange >= min_fold & padj <= max_p)) %>%
        filter(any(elapsed == hours[i+1] & log2foldchange >= min_fold & padj <= max_p)) %>%
        filter(any(elapsed == hours[i+2] & log2foldchange >= min_fold & padj <= max_p)) %>%
        filter(!any(!(elapsed %in% conditions) & log2foldchange >= min_fold & padj <= max_p)) %>%
        ungroup() %>%
        select(id) %>%
        distinct(id) %>%
        left_join(d, by = "id") %>%
        write_tsv(output_file)

    ## down-regulated in two conditions (< -2), and stable or
    ## up-regulated in all other conditions !(< -2)
    output_file <- sub(".txt",
                    paste0("_downregulated_", hours[i], "-",
                        hours[i+1], "-", hours[i+2], "h_only.tsv"),
                    input_file)
    d1 %>%
        filter(any(elapsed == hours[i] & log2foldchange <= -min_fold & padj <= max_p)) %>%
        filter(any(elapsed == hours[i+1] & log2foldchange <= -min_fold & padj <= max_p)) %>%
        filter(any(elapsed == hours[i+2] & log2foldchange <= -min_fold & padj <= max_p)) %>%
        filter(!any(!(elapsed %in% conditions) & log2foldchange <= -min_fold & padj <= max_p)) %>%
        ungroup() %>%
        select(id) %>%
        distinct(id) %>%
        left_join(d, by = "id") %>%
        write_tsv(output_file)
}
```

```
## ************************************************************************ ##
##         ##
##           quadruple condition: up- and down-regulated          ##
##         ##
## ************************************************************************ ##

## select genes that are up-regulated in four consecutive conditions,
## and stable or down-regulated in the other condition

for (i in seq(1, length(hours) - 3)) {
  conditions <- c(hours[i], hours[i+1], hours[i+2], hours[i+3])
  ## up-regulated
  output_file <- sub(".txt",
              paste0("_upregulated_", hours[i], "-",
                  hours[i+1], "-", hours[i+2], "-",
                  hours[i+3], "h_only.tsv"),
              input_file)
  d1 %>%
    filter(any(elapsed == hours[i] & log2foldchange >= min_fold & padj <= max_p)) %>%
    filter(any(elapsed == hours[i+1] & log2foldchange >= min_fold & padj <= max_p)) %>%
    filter(any(elapsed == hours[i+2] & log2foldchange >= min_fold & padj <= max_p)) %>%
    filter(any(elapsed == hours[i+3] & log2foldchange >= min_fold & padj <= max_p)) %>%
    filter(!any(!(elapsed %in% conditions) & log2foldchange >= min_fold & padj <= max_p)) %>%
    ungroup() %>%
    select(id) %>%
    distinct(id) %>%
    left_join(d, by = "id") %>%
    write_tsv(output_file)

  ## down-regulated in two conditions (< -2), and stable or
  ## up-regulated in all other conditions !(< -2)
  output_file <- sub(".txt",
              paste0("_downregulated_", hours[i],
                  hours[i+1], "-", hours[i+2], "-",
                  hours[i+3], "h_only.tsv"),
              input_file)
  d1 %>%
    filter(any(elapsed == hours[i] & log2foldchange <= -min_fold & padj <= max_p)) %>%
    filter(any(elapsed == hours[i+1] & log2foldchange <= -min_fold & padj <= max_p)) %>%
    filter(any(elapsed == hours[i+2] & log2foldchange <= -min_fold & padj <= max_p)) %>%
    filter(any(elapsed == hours[i+3] & log2foldchange <= -min_fold & padj <= max_p)) %>%
    filter(!any(!(elapsed %in% conditions) & log2foldchange <= -min_fold & padj <= max_p)) %>%
    ungroup() %>%
    select(id) %>%
    distinct(id) %>%
    left_join(d, by = "id") %>%
    write_tsv(output_file)
}


## ************************************************************************ ##
##         ##
##           quintuple condition: up- and down-regulated          ##
##         ##
## ************************************************************************ ##

## select genes that are up-regulated in all conditions

## up-regulated
output_file <- sub(".txt", "_upregulated_all_conditions.tsv", input_file)
i <- 1
d1 %>%
  filter(any(elapsed == hours[i] & log2foldchange >= min_fold & padj <= max_p)) %>%
```

```
        filter(any(elapsed == hours[i+1] & log2foldchange >= min_fold & padj <= max_p)) %>%
        filter(any(elapsed == hours[i+2] & log2foldchange >= min_fold & padj <= max_p)) %>%
        filter(any(elapsed == hours[i+3] & log2foldchange >= min_fold & padj <= max_p)) %>%
        filter(any(elapsed == hours[i+4] & log2foldchange >= min_fold & padj <= max_p)) %>%
        ungroup() %>%
        select(id) %>%
        distinct(id) %>%
        left_join(d, by = "id") %>%
        write_tsv(output_file)

    ## down-regulated
    output_file <- sub(".txt", "_downregulated_all_conditions.tsv", input_file)
    i <- 1
    d1 %>%
        filter(any(elapsed == hours[i] & log2foldchange <= -min_fold & padj <= max_p)) %>%
        filter(any(elapsed == hours[i+1] & log2foldchange <= -min_fold & padj <= max_p)) %>%
        filter(any(elapsed == hours[i+2] & log2foldchange <= -min_fold & padj <= max_p)) %>%
        filter(any(elapsed == hours[i+3] & log2foldchange <= -min_fold & padj <= max_p)) %>%
        filter(any(elapsed == hours[i+4] & log2foldchange <= -min_fold & padj <= max_p)) %>%
        ungroup() %>%
        select(id) %>%
        distinct(id) %>%
        left_join(d, by = "id") %>%
        write_tsv(output_file)
}
```
