# Supplementary material for: Transcriptome Profiles of Nod Factor-independent Symbiosis in the Tropical Legume Aeschynomene evenia
Source: Sci Rep. 2018 Jul 19;8:10934. doi: 10.1038/s41598-018-29301-0 (PMC6053390; doi:10.1038/s41598-018-29301-0)

Supplementary Figure 1

Transcriptome Profiles of Nod Factor-independent Symbiosis in the Tropical Legume *Aeschynomene evenia*

Djamel Gully, Pierre Czernic, Stéphane Cruveiller, Frédéric Mahé, Cyrille Longin, David Vallenet, Philippe François, Sabine Nidelet, Stéphanie Rialle, Eric Giraud, Jean-François Arrighi, Maitrayee Das Gupta and Fabienne Cartieaux

Supplementary Figure 1: Heatmap of DEGs throughout nodulation kinetics. Red indicates up-regulation and green indicates down-regulation. Chroma color from green to red indicates Log2 (fold change) from less to more. Clustering was performed with the MultiExperiment Viewer 4.8 by hierarchical clustering of all genes and samples using Manhattan distance calculations.

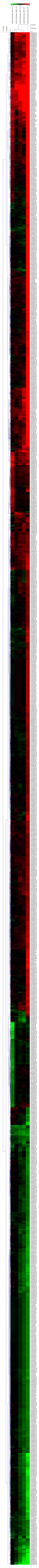

Supplement: Supplementary file 2 — Supplementary Figure 1 [file 41598_2018_29301_MOESM2_ESM.pdf]
