# Supplementary material for: Transcriptome Profiles of Nod Factor-independent Symbiosis in the Tropical Legume Aeschynomene evenia
Source: Sci Rep. 2018 Jul 19;8:10934. doi: 10.1038/s41598-018-29301-0 (PMC6053390; doi:10.1038/s41598-018-29301-0)
Supplement: Supplementary file 4 — Supplementary Figure 3 [file 41598_2018_29301_MOESM4_ESM.pdf]

Supplementary Figure 3

Transcriptome Profiles of Nod Factor-independent Symbiosis in the Tropical Legume *Aeschynomene evenia*

Djamel Gully, Pierre Czernic, Stéphane Cruveiller, Frédéric Mahé, Cyrille Longin, David Vallenet, Philippe François, Sabine Nidelet, Stéphanie Rialle, Eric Giraud, Jean-François Arrighi, Maitrayee Das Gupta and Fabienne Cartieaux

Supplementary Figure 3: Heatmap of differentially expressed symbiotic genes throughout nodulation kinetics (A). Red indicates up-regulation and green indicates down-regulation. Chroma color from green to red indicates Log2 (fold change) from less to more. The Log2 values for each time points are presented in the table (B).

A

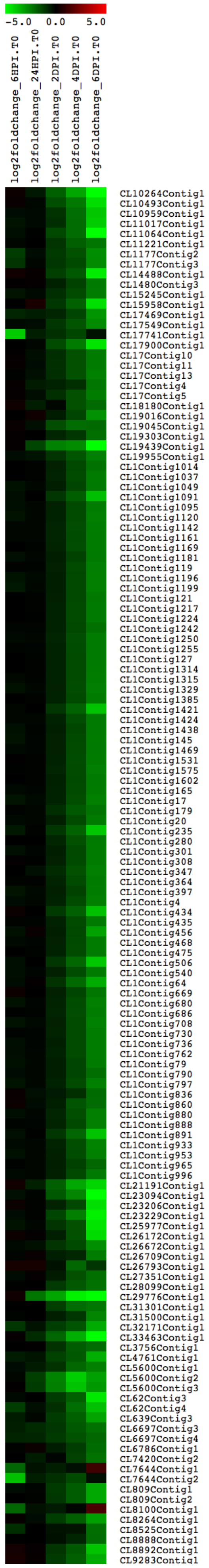

B

Contig(s)	Blast definition
CL10264Contig1	Structural constituent of cell wall, putative OS=Ricinus communis GN=RCOM_0380590 PE=4 SV=1
CL10493Contig1	Polygalacturonase OS=Medicago truncatula GN=MTR_2g103650 PE=4 SV=1
CL10959Contig1	Pectinesterase OS=Glycine max PE=3 SV=1
CL11017Contig1	Pectinesterase OS=Glycine max GN=Gma.52091 PE=3 SV=1
CL11064Contig1	Proline-rich protein OS=Medicago truncatula GN=MTR_4g108150 PE=2 SV=1
CL11221Contig1	Casparian strip membrane protein 5 OS=Glycine max PE=2 SV=1
CL1177Contig2	Pectinesterase OS=Glycine max PE=3 SV=1
CL1177Contig3	Pectinesterase OS=Medicago truncatula GN=MTR_8g104630 PE=3 SV=1
CL14488Contig1	Peroxidase family protein OS=Arabidopsis lyrata subsp. lyrata GN=ARALYDRAFT_489264 PE=3 SV=1
CL1480Contig3	Xyloglucan endotransglycosylase OS=Medicago truncatula GN=MTR_2g095800 PE=4 SV=1
CL15245Contig1	Pectinesterase (Fragment) OS=Glycine max PE=3 SV=1
CL15958Contig1	Structural constituent of cell wall, putative OS=Ricinus communis GN=RCOM_0380590 PE=4 SV=1
CL17469Contig1	Laccase-15 OS=Medicago truncatula GN=MTR_3g101640 PE=4 SV=1
CL17549Contig1	Pectinesterase (Fragment) OS=Solanum lycopersicum PE=2 SV=1
CL17741Contig1	Polygalacturonase (Precursor) OS=Glycine max GN=PG7 PE=2 SV=1
CL17900Contig1	Pectate lyase 1-27 OS=Medicago truncatula GN=MTR_7g059290 PE=4 SV=1
CL17Contig10	Peroxidase OS=Medicago truncatula GN=MTR_2g029830 PE=3 SV=1
CL17Contig11	Peroxidase OS=Glycine max GN=PRX3 PE=2 SV=1
CL17Contig13	Peroxidase OS=Medicago truncatula GN=MTR_2g029740 PE=3 SV=1
CL17Contig4	Peroxidase OS=Medicago truncatula GN=MTR_2g029730 PE=3 SV=1
CL17Contig5	Peroxidase OS=Medicago truncatula GN=MTR_2g029800 PE=3 SV=1
CL18180Contig1	Laccase 110b OS=Populus trichocarpa GN=LAC110b PE=4 SV=1
CL19016Contig1	Peroxidase OS=Medicago truncatula GN=MTR_118s0001 PE=3 SV=1
CL19045Contig1	Expansin OS=Glycine max PE=2 SV=1
CL19303Contig1	Cinnamoyl-CoA reductase, putative OS=Ricinus communis GN=RCOM_0180630 PE=4 SV=1
CL19439Contig1	Pistil-specific extensin-like protein OS=Medicago truncatula GN=MTR_7g102770 PE=4 SV=1
CL19955Contig1	Pectinesterase OS=Glycine max PE=3 SV=1
CL1Contig1014	L.esculentum extensin (class I) gene, complete cds
CL1Contig1037	L.esculentum extensin (class I) gene, complete cds
CL1Contig1049	P.vulgaris hydroxyproline-rich glycoprotein (HRGP) mRNA, exon 2
CL1Contig1091	Hydroxyproline-rich glycoprotein (HRGP) (Fragment) OS=Phaseolus vulgaris PE=2 SV=1
CL1Contig1095	Hydroxyproline-rich glycoprotein (HRGP) (Fragment) OS=Phaseolus vulgaris PE=2 SV=1
CL1Contig1120	P.vulgaris hydroxyproline-rich glycoprotein (HRGP) mRNA, exon 2
CL1Contig1142	L.esculentum extensin (class I) gene, complete cds
CL1Contig1161	L.esculentum extensin (class I) gene, complete cds
CL1Contig1169	L.esculentum extensin (class I) gene, complete cds
CL1Contig1181	Hydroxyproline-rich glycoprotein (HRGP) (Fragment) OS=Phaseolus vulgaris PE=2 SV=1
CL1Contig119	L.esculentum extensin (class I) gene, complete cds
CL1Contig1196	L.esculentum extensin (class I) gene, complete cds
CL1Contig1199	P.vulgaris hydroxyproline-rich glycoprotein (HRGP) mRNA, exon 2
CL1Contig121	L.esculentum extensin (class I) gene, complete cds
CL1Contig1217	L.esculentum extensin (class I) gene, complete cds
CL1Contig1224	L.esculentum extensin (class I) gene, complete cds
CL1Contig1242	L.esculentum extensin (class I) gene, complete cds
CL1Contig1250	L.esculentum extensin (class I) gene, complete cds
CL1Contig1255	L.esculentum extensin (class I) gene, complete cds
CL1Contig127	L.esculentum extensin (class I) gene, complete cds
CL1Contig1314	L.esculentum extensin (class I) gene, complete cds
CL1Contig1315	L.esculentum extensin (class I) gene, complete cds
CL1Contig1329	P.vulgaris hydroxyproline-rich glycoprotein (HRGP) mRNA, exon 2
CL1Contig1385	L.esculentum extensin (class I) gene, complete cds
CL1Contig1421	Hydroxyproline-rich glycoprotein (HRGP) (Fragment) OS=Phaseolus vulgaris PE=2 SV=1
CL1Contig1424	P.vulgaris hydroxyproline-rich glycoprotein (HRGP) mRNA, exon 2
CL1Contig1438	L.esculentum extensin (class I) gene, complete cds
CL1Contig145	P.vulgaris hydroxyproline-rich glycoprotein (HRGP) mRNA, exon 2
CL1Contig1469	P.vulgaris hydroxyproline-rich glycoprotein (HRGP) mRNA, exon 2
CL1Contig1531	L.esculentum extensin (class I) gene, complete cds
CL1Contig1575	L.esculentum extensin (class I) gene, complete cds
CL1Contig1602	L.esculentum extensin (class I) gene, complete cds
CL1Contig165	L.esculentum extensin (class I) gene, complete cds
CL1Contig17	P.vulgaris hydroxyproline-rich glycoprotein (HRGP) mRNA, exon 2
CL1Contig179	Phaseolus vulgaris cell wall type 2 proline rich protein PvPRP2-37 (Pvprp2-37) mRNA, partial cds
CL1Contig20	L.esculentum extensin (class I) gene, complete cds
CL1Contig235	P.vulgaris hydroxyproline-rich glycoprotein (HRGP) mRNA, 3' end
CL1Contig280	L.esculentum extensin (class I) gene, complete cds
CL1Contig301	L.esculentum extensin (class I) gene, complete cds
CL1Contig308	L.esculentum extensin (class I) gene, complete cds
CL1Contig347	L.esculentum extensin (class I) gene, complete cds
CL1Contig364	L.esculentum extensin (class I) gene, complete cds
CL1Contig397	L.esculentum extensin (class I) gene, complete cds
CL1Contig4	L.esculentum extensin (class I) gene, complete cds
CL1Contig434	L.esculentum extensin (class I) gene, complete cds
CL1Contig435	L.esculentum extensin (class I) gene, complete cds
CL1Contig456	Peroxidase OS=Medicago truncatula GN=MTR_118s0001 PE=3 SV=1
CL1Contig468	L.esculentum extensin (class I) gene, complete cds
CL1Contig475	L.esculentum extensin (class I) gene, complete cds
CL1Contig506	P.vulgaris hydroxyproline-rich glycoprotein (HRGP) mRNA, exon 2
CL1Contig540	P.vulgaris hydroxyproline-rich glycoprotein (HRGP) mRNA, exon 2
CL1Contig64	Proline-rich protein OS=Glycine max GN=PRP PE=2 SV=1
CL1Contig669	L.esculentum extensin (class I) gene, complete cds
CL1Contig680	P.vulgaris hydroxyproline-rich glycoprotein (HRGP) mRNA, exon 2
CL1Contig686	L.esculentum extensin (class I) gene, complete cds
CL1Contig708	P.vulgaris hydroxyproline-rich glycoprotein (HRGP) mRNA, exon 2
CL1Contig730	L.esculentum extensin (class I) gene, complete cds
CL1Contig736	P.vulgaris hydroxyproline-rich glycoprotein (HRGP) mRNA, exon 2
CL1Contig762	P.vulgaris hydroxyproline-rich glycoprotein (HRGP) mRNA, exon 2
CL1Contig79	Hydroxyproline-rich glycoprotein (HRGP) (Fragment) OS=Phaseolus vulgaris PE=2 SV=1
CL1Contig790	L.esculentum extensin (class I) gene, complete cds
CL1Contig797	P.vulgaris hydroxyproline-rich glycoprotein (HRGP) mRNA, exon 2
CL1Contig836	L.esculentum extensin (class I) gene, complete cds
CL1Contig860	L.esculentum extensin (class I) gene, complete cds
CL1Contig880	P.vulgaris hydroxyproline-rich glycoprotein (HRGP) mRNA, exon 2
CL1Contig888	L.esculentum extensin (class I) gene, complete cds
CL1Contig891	Hydroxyproline-rich glycoprotein (HRGP) (Fragment) OS=Phaseolus vulgaris PE=2 SV=1
CL1Contig933	L.esculentum extensin (class I) gene, complete cds
CL1Contig953	P.vulgaris hydroxyproline-rich glycoprotein (HRGP) mRNA, exon 2
CL1Contig965	L.esculentum extensin (class I) gene, complete cds
CL1Contig996	P.vulgaris hydroxyproline-rich glycoprotein (HRGP) mRNA, 3' end
CL21191Contig1	Pectinesterase OS=Glycine max PE=3 SV=1
CL23094Contig1	Expansin OS=Glycine max GN=EXP1 PE=2 SV=1
CL23206Contig1	Peroxidase OS=Medicago truncatula GN=MTR_118s0001 PE=3 SV=1
CL23229Contig1	PREDICTED: probable pectate lyase 15-like [Glycine max]
CL25977Contig1	Peroxidase (Fragment) OS=Medicago truncatula GN=MTR_118s0020 PE=3 SV=1
CL26172Contig1	Peroxidase OS=Medicago truncatula GN=MTR_118s0001 PE=3 SV=1
CL26672Contig1	Pectinesterase OS=Glycine max GN=Gma.20242 PE=3 SV=1
CL26709Contig1	Chalcone--flavonone isomerase 2 OS=Lotus japonicus GN=CHI2 PE=2 SV=1
CL26793Contig1	Pectinesterase OS=Glycine max PE=3 SV=1
CL27351Contig1	Casparian strip membrane protein 1 OS=Glycine max PE=2 SV=1
CL28099Contig1	Endo-1,4-beta-glucanase (Precursor) OS=Glycine max GN=Cel7 PE=2 SV=1
CL29776Contig1	Pectate lyase, putative OS=Ricinus communis GN=RCOM_0903690 PE=4 SV=1
CL31301Contig1	Beta-galactosidase OS=Glycine max GN=Gma.12808 PE=3 SV=1
CL31500Contig1	Peroxidase OS=Medicago truncatula GN=MTR_5g074710 PE=3 SV=1
CL32171Contig1	Pectinesterase OS=Medicago truncatula GN=MTR_3g008640 PE=3 SV=1
CL33463Contig1	Pectinesterase (Fragment) OS=Glycine max PE=3 SV=1
CL3756Contig1	Glucan endo-1,3-beta-glucosidase OS=Glycine max PE=1 SV=1
CL4761Contig1	Expansin-like protein OS=Medicago truncatula GN=MTR_5g013440 PE=3 SV=1
CL5600Contig1	Peroxidase OS=Medicago truncatula GN=MTR_7g072510 PE=3 SV=1
CL5600Contig2	Cationic peroxidase 1 OS=Arachis hypogaea GN=PNC1 PE=1 SV=2
CL5600Contig3	Peroxidase OS=Medicago truncatula GN=MTR_7g072510 PE=3 SV=1
CL62Contig3	14 kDa proline-rich protein DC2.15 OS=Medicago truncatula GN=MTR_4g101280 PE=2 SV=1
CL62Contig4	14 kDa proline-rich protein DC2.15 OS=Medicago truncatula GN=MTR_4g101280 PE=2 SV=1
CL639Contig3	Expansin-like protein OS=Medicago truncatula GN=MTR_5g013440 PE=3 SV=1
CL6697Contig3	Beta-fructofuranosidase, insoluble isoenzyme CWINV1 OS=Medicago truncatula GN=MTR_1g015910 PE=3 SV=1
CL6697Contig4	Beta-fructofuranosidase, insoluble isoenzyme CWINV1 OS=Medicago truncatula GN=MTR_1g015910 PE=3 SV=1
CL6786Contig1	Peroxidase OS=Medicago truncatula GN=MTR_5g074970 PE=3 SV=1
CL7420Contig2	Laccase OS=Medicago truncatula GN=MTR_046s0015 PE=4 SV=1
CL7644Contig1	Polygalacturonase OS=Medicago truncatula GN=MTR_2g032710 PE=4 SV=1
CL7644Contig2	Polygalacturonase OS=Medicago truncatula GN=MTR_2g032710 PE=4 SV=1
CL809Contig1	Peroxidase (Precursor) OS=Glycine max GN=sEPa1 PE=2 SV=1
CL809Contig2	Peroxidase OS=Medicago truncatula GN=MTR_2g088770 PE=3 SV=1
CL8100Contig1	Polygalacturonase (Precursor) OS=Glycine max GN=PG7 PE=2 SV=1
CL8264Contig1	Pectate lyase 1-27 OS=Medicago truncatula GN=MTR_7g059290 PE=4 SV=1
CL8525Contig1	Polygalacturonase PG1 OS=Glycine max GN=Gma.57550 PE=2 SV=1
CL8888Contig1	Pectate lyase OS=Medicago truncatula GN=MTR_3g086310 PE=4 SV=1
CL8892Contig1	Structural constituent of cell wall, putative OS=Ricinus communis GN=RCOM_0155160 PE=4 SV=1
CL9283Contig1	Peroxidase OS=Medicago truncatula GN=MTR_1g115900 PE=3 SV=1
