# Supplementary material for: Transcriptome Profiles of Nod Factor-independent Symbiosis in the Tropical Legume Aeschynomene evenia
Source: Sci Rep. 2018 Jul 19;8:10934. doi: 10.1038/s41598-018-29301-0 (PMC6053390; doi:10.1038/s41598-018-29301-0)
Supplement: Supplementary file 6 — Supplementary Table 2 [file 41598_2018_29301_MOESM6_ESM.pdf]

Supplementary Table 2

Transcriptome Profiles of Nod Factor-independent Symbiosis in the Tropical Legume *Aeschynomene evenia*

Djamel Gully, Pierre Czernic, Stéphane Cruveiller, Frédéric Mahé, Cyrille Longin, David Vallenet, Philippe François, Sabine Nidelet, Stéphanie Rialle, Eric Giraud, Jean-François Arrighi, Maitrayee Das Gupta and Fabienne Cartieaux

Supplementary Table 2: List of symbiotic genes reported in Figure 4.

Supplementary Table 2: List of symbiotic genes reported in Figure 4.

Genes in model legumes	Aeschynomene contigs	Medicago truncatula	reference	Lotus japonicus	reference
MtLYK3, LjNFR1	no hit	AY372406.1	Limpens et al. 2003	AJ575249.1	Radutoiu et al. 2003
MtNFP, LjNFR5	CL28443Contig1	DQ496250.1	Arrighi et al. 2006	AJ575255.1	Madsen et al. 2003
MtLYK4, LjLYS2	no hit	BN001117.1	Limpens et al. 2003	AB506699.1	Lohmann et al. 2010
MtLYR3, LjLYS12	no hit	XM_003611842.2	Arrighi et al. 2006	AB506702.1	Lohmann et al. 2010
MtDMI2, LjSYMRK	CL3159Contig1	AJ418368.1	Endre et al. 2002	AF492655.1	Stracke et al. 2002
MtSYMREM1	CL8816Contig3	JQ061257.1	Lefebvre et al. 2010	N.A	
LjCASTOR	CL11378Contig2	N.A		AB162157.1	Imaizumi-Anraku et al. 2005
MtDMI1, LjPOLLUX	CL15061Contig1	AY497771	Ané et al. 2004	AB162158.1	Imaizumi-Anraku et al. 2005
LjNUP133	CL11257Contig1	N.A		AJ890251.1	Kanamori et al. 2006
LjNUP85	CL4854Contig1	N.A		AB284835.1	Saito et al. 2007
LjNENA	CL9324Contig2	N.A		AB506696.1	Groth et al. 2010
MtDMI3, LjCCaMK	CL4754Contig2	AY502066.1	Lévy et al. 2004	AM230793.1	Tirichine et al. 2006
LjSIP1	CL7054Contig3	N.A		EU559710.2	Zhu et al. 2008
LjSIP2	CL1713Contig1	N.A		HQ910409.2	Chen et al. 2012
MtNSP1, LjNSP1	CL11061Contig1	AJ972478	Smit et al. 2005	EF017372.1	Heckmann et al. 2006
MtNSP2, LjNSP2	CL16416Contig1	AJ832138	Kalo et al. 2005	DQ665943.1	Heckmann et al. 2006
LjERF1	CL16441Contig1	N.A		AB378626.1	Asamizu et al. 2008
MtNIN, LjNIN	CL1442Contig1	FJ719774.1	Marsh et al. 2007	AJ239041.1	Schauser et al. 1999
MtCRE1, LjHK1	CL2164Contig1	XM_003630966.2	Gonzalez-Rizzo et al. 2006	AM287033.1	Murray et al. 2007
MtNOOT	CL4478Contig1	JN180859.1	Couzigou et al. 2012	N.A	
MtbHLH476	CL23562Contig1	XP.003611493.1	Ariel et al. 2012	N.A	
MtNIP/LATD	CL14509Contig1	GQ401665.1	Yendrek et al. 2010	N.A	
MtIPD3, LjCYCLOPS	CL9907Contig1	EF117279.1	Messinese et al. 2007	EF569221.1	Yano et al. 2008
MtLYK10, LjLYS3 (LjEPR3)	no hit	XM_003613117.2	Arrighi et al. 2006	BAI79284.1	Lohmann et al. 2010
MtVPY	CL8739Contig1	GQ423209.1	Murray et al. 2011	N.A	
LjARPC1	CL8613Contig2	N.A		JX446368.1	Hossain et al. 2012
MtNAP1, LjNAP1	CL5870Contig1	HM590708	Miyahara et al. 2010	AM946365.1	Yokota et al. 2009
LjPIR1	CL3526Contig2	N.A		AM946364.1	Yokota et al. 2009
MtLIN, LjCERBERUS	CL5867Contig1	EU926660.1	Kiss et al. 2009	AB505797.1	Yano et al. 2009
MtPUB1	CL25436Contig1	DAA33939.1	Mbengue et al. 2010	N.A	
MtRPG	no hit	DQ854741.1	Arrighi et al. 2008	N.A	
MtANN1	no hit	Y15036	de Carvalho Niebel et al. 1998	N.A	
LjLNP	CL3644Contig1	N.A		AF156780.1	Roberts et al. 1999
LjnsRING	CL2048Contig2	N.A		AB272096	Shimomura et al. 2006
MtMMPL1	no hit	Y18249.2	Combier et al. 2007	N.A	
MtFLOT2	no hit	ADA83095.1	Haney and Long 2010	N.A	
MtFLOT4	no hit	ADA83097.1	Haney and Long 2010	N.A	
MtSUNN, LjHAR1	CL14519Contig1	AY769943	Schnabel et al. 2005	AB092810.1	Nishimura et al. 2002
LjASTRAY	CL3682Contig1	N.A		AB092677.1	Nishimura et al. 2002
LjKLAVIER	CL11489Contig1	N.A		AB675946.1	Miyazawa et al. 2010
MtEFD	CL16720Contig1	EU251063.1	Vernié et al. 2008	N.A	
MtSICKLE	CL4710Contig1	EU709495.1	Penmetsa et al. 2008	N.A	
MtRDN1	CL5200Contig3	GU580937	Schnabel et al. 2011	N.A	
LjIGN1	CL3704Contig1	N.A		AB251640	Kumagai et al. 2007
MtDNF1	CL5297Contig1	XP_003626310.1	Wang et al. 2010	N.A	
LjSST1	CL81Contig3	N.A		AM283536.1	Krusell et al. 2005
MtSymCRK	no hit	AES71610.2	Berrabah et al. 2014	N.A	
LjSUNERGOS1	no hit	N.A		KJ671531.1	Yoon et al. 2014
LjVAG1	no hit	N.A		AB871650.1	Suzaki et al. 2014
MtRSD	CL7810Contig2	JX307863.1	Sinharoy et al. 2013	N.A	
LjSEN1	CL2022Contig1	N.A		AB573230.1	Hakoyama et al. 2012
MtDNF2	no hit	XM_003607900.2	Bourcy et al. 2013	AB167409.1	Kinoshita et al. 2004
