# Supplementary material for: Transcriptome Profiles of Nod Factor-independent Symbiosis in the Tropical Legume Aeschynomene evenia
Source: Sci Rep. 2018 Jul 19;8:10934. doi: 10.1038/s41598-018-29301-0 (PMC6053390; doi:10.1038/s41598-018-29301-0)
Supplement: Supplementary file 7 — Supplementary Table 3 [file 41598_2018_29301_MOESM7_ESM.pdf]

Supplementary Table 3

Transcriptome Profiles of Nod Factor-independent Symbiosis in the Tropical Legume *Aeschynomene evenia*

Djamel Gully, Pierre Czernic, Stéphane Cruveiller, Frédéric Mahé, Cyrille Longin, David Vallenet, Philippe François, Sabine Nidelet, Stéphanie Rialle, Eric Giraud, Jean-François Arrighi, Maitrayee Das Gupta and Fabienne Cartieaux

Supplementary Table 3: Primers used in qPCR validation.

Supplementary Table 3: Primers used in qPCR validation.

Contig in A. evenia serrulata RNAseq	Blast definition	Forward primer	Reverse primer
CL27076Contig1	Nodulin-related integral membrane protein DUF125 OS=Medicago truncatula GN=MTR_6g072155	GCAGCAGTTCTGGGAGCAAATG	TGGTCCTCACATCCTTCCTTACG
CL20994Contig1	Copper transport protein atox1, putative OS=Ricinus communis	AAAGGTGACAGTGAATGGTTGGG	GTTATAGGACGATGAAGGCTGAGG
CL20199Contig1	Auxin-induced protein AUX28 OS=Glycine max	ACCAACACCTCTCTGATTCCTTAG	ACAAGCATCCAGTCACCATCC
CL144Contig2	Late elongated hypocotyl and circadian clock associated-1-like protein 2 OS=Glycine max	AGTTGGAGAGTTCTGGTGCTTC	CTTGGGTAATTCTGATTGCCTTGC
CL2184Contig2	NADP-dependent glyceraldehyde-3-phosphate dehydrogenase OS=Medicago truncatula	AAATAGCAAAGCCAGCCAAAGATG	CCCTCTCCAAGAAGCCTTACCC
CL27719Contig1	Granule bound starch synthase Ib OS=Lotus japonicus	ACTGCCCTTATCCCTTGCTACTTG	CTGCGAATGCGAATCTTCCTTGG
CL22113Contig1	Granule-bound starch synthase OS=Glycine max	TGGTGGTTTAGGAGATGTTCTTGG	CGTGGTGCGATGGTCATAACTC
CL7644Contig2	Polygalacturonase OS=Medicago truncatula GN=MTR_2g032710	GCCAAGGAGTGCTAATCAATGAAG	GGACCACAAGTTACACCATCAATG
CL22393Contig1	Chaperonin-like RbcX protein OS=Arabidopsis thaliana GN=At4g04330	CCTTGTGCCTACCCTTCATTGC	GTGTAAGCGTGTGGATTGAGAGC
CL513Contig1	Inositol methyltransferase OS=Glycine max GN=Gma.31658	GCAGGAAGGCAATACCAGAGAAG	GCATAGCCAGTGTCATCAAATAGC
CL433Contig1	Subtilisin-like serine protease OS=Medicago truncatula GN=MTR_5g011190	TTGAACTATCCTTCCTTCTCTGTC	CACACCAACATTTACACCCTCTG
CL9900Contig1	Transcription factor C3H OS=Lotus japonicus GN=RING-G83	GCCTGAACCAAGAGACCATCG	GGAACTCGCTCAAGCACACC
CL4595Contig1	Sulfate/bicarbonate/oxalate exchanger and transporter sat-1 OS=Medicago truncatula GN=MTR_3g087730	GGCACAACTAAGTCCTGAATATGG	ATTGAGGCAAGAAGCATTGAATCC
CL1460Contig2	Universal stress protein OS=Arachis hypogaea	TCTTCTCTACGCCAAACCCTTTCC	TTCCACCACCACCTCATCAACC
CL5726Contig2	AeNCR24	TCATGGTCAAGTGGCTGGTG	GCAATCTCGCATACAATCATAGG
CL3Contig3	Nonsymbiotic hemoglobin OS=Glycine max	AGTGCTTCCGATGTGGGTCTC	AACGGTGGAATGTGACTTGAGC
CL1632Contig2	Auxin-induced protein 5NG4 OS=Medicago truncatula GN=MTR_2g102340	CCTATCGCTTCATCTTCGCATCG	GCCACCGCTTCCACAAACAG
CL27Contig3	Elongation factor 1-alpha	AATGGTGATGCTGGTATGGTTAAG	TCTTCTTCTGTGCTGCCTTGG
